# Boosting Sensitivity, Stability, and Speed: A Polydopamine-Engineered Silver Nanoparticle Lateral Flow Immunoassay for Aflatoxin B_1_ in Maize

**DOI:** 10.3390/toxins18030129

**Published:** 2026-03-03

**Authors:** Xinge Mo, Shuhong Zhang, Zixuan He, Xiaoyang Li, Xiangmin Li, Yonghua Xiong, Hu Jiang

**Affiliations:** 1 State Key Laboratory of Food Science and Resources, Nanchang University, Nanchang 330047, China; xinge0529@163.com (X.M.); redtreetree@163.com (S.Z.); box123124@163.com (Z.H.); xiongyonghua@ncu.edu.cn (Y.X.); 2 School of Food Science and Technology, Nanchang University, Nanchang 330047, China; 3 College of Life Science and Technology, Beijing University of Chemical Technology, Beijing 100029, China; xyli@ncu.edu.cn; 4 Sino German Joint Research Institute, Nanchang University, Nanchang 330047, China; lixiangmin73@163.com

**Keywords:** lateral flow immunoassay, polydopamine-coated silver nanoparticles, aflatoxin B_1_, maize

## Abstract

Conventional colorimetric lateral flow immunoassays (LFIAs) often suffer from insufficient sensitivity for detecting trace low-molecular-weight contaminants like mycotoxins. The development of colorimetric probes with a high molar extinction coefficient is therefore critical for enhancing detection performance. Although silver nanoparticles (AgNPs) exhibit an extremely high molar extinction coefficient, their practical application in LFIA is hindered by inherent chemical instability and suboptimal visual contrast. To address these limitations, we have engineered robust and high-performance polydopamine-functionalized AgNPs (Ag@PDA NPs) as advanced LFIA signal probes, which were successfully used for detecting aflatoxin B_1_ (AFB_1_) in maize. The multifunctional PDA nanoshell effectively shields the Ag core from oxidation and other destabilizing factors, ensuring superior long-term stability and significantly enhancing colorimetric contrast. Moreover, it improves the colloidal hydrophilicity, enabling faster and more uniform migration kinetics along the test strip. Leveraging these engineered properties, the developed assay achieved a limit of detection (LOD) of 0.23 ng mL^−1^ for AFB_1_ in buffer, representing a remarkable 2.17-fold sensitivity enhancement over conventional colloidal gold-based LFIAs. Validation in spiked maize samples confirmed high reliability, with recoveries ranging from 95.70% to 119.28% and precision (inter-/intra-assay CVs) below 13.03%.

## 1. Introduction

Aflatoxin B_1_ (AFB_1_), a toxic metabolite produced by *Aspergillus* species [1], readily contaminates maize and maize products [2]. AFB_1_ is highly toxic and carcinogenic, with widespread distribution [3]. Long-term ingestion of AFB_1_-contaminated maize poses a severe hazard to human health [4]. The International Agency for Research on Cancer (IARC) has classified AFB_1_ as a Class 1A carcinogen. Therefore, the development of a rapid, sensitive, and accurate AFB_1_ detection method is in urgent demand. Currently, the primary detection methods for AFB_1_ include high-performance liquid chromatography (HPLC) [5,6], liquid chromatography–tandem mass spectrometry (LC-MS/MS) [7,8], and enzyme-linked immunosorbent assay (ELISA) [9,10]. However, these techniques often involve complex procedures and require sophisticated instrumentation and trained personnel, which confines testing to centralized laboratories and renders them unsuitable for the rapid on-site screening necessary for timely food safety management. Consequently, lateral flow immunoassay (LFIA) has emerged as the preferred alternative for point-of-care testing, offering an ideal balance of operational simplicity, rapidity, and cost-effectiveness [11,12]. Yet, this practical advantage is often offset by insufficient analytical sensitivity in traditional colloidal gold-based formats [13,14], which restricts its application in detecting trace levels of AFB_1_ in complex grain matrices such as maize.

This sensitivity bottleneck is largely attributed to the inherent optical limitations of conventional colloidal gold probes. While stable and easy to prepare, their molar extinction coefficients are often insufficient to generate intense visual signals at trace analyte concentrations, necessitating the exploration of alternative high-performance nanomaterials. To address this, silver nanoparticles (AgNPs) have attracted significant attention. Compared to colloidal gold, AgNPs exhibit substantially higher molar extinction coefficients due to stronger surface plasmon resonance effects [15,16], theoretically offering much greater signal amplification potential. However, the practical application of bare AgNPs in visual LFIAs is often hampered by two critical limitations: (1) their distinct bright yellow color, which provides poor visual contrast against white nitrocellulose membranes compared to the deep red of colloidal gold; and (2) their relatively poor chemical stability (e.g., susceptibility to oxidation and light environments) [17], which can negatively impact the performance of immunoprobes. To overcome these limitations, surface modification with polydopamine (PDA) was employed. Dopamine (DA) is a small molecule containing an arene and an amino group that undergoes self-polymerization under mild conditions (such as a weakly alkaline environment) to form PDA films on various material surfaces through both non-covalent and covalent bonds [18,19]. This PDA coating strategy is strategically employed here to address the limitations of bare AgNPs. Mechanistically, the formation of a robust PDA shell serves a triple function: (1) it acts as a protective barrier, effectively shielding the highly reactive Ag core from oxidation and significantly improving chemical stability [20,21]; (2) its hydrophilic nature and steric hindrance reduce particle-to-particle aggregation and nonspecific adsorption on the test strip; and (3) its surface is rich in abundant functional groups (e.g., catechol and amine groups) that facilitate high-efficiency, covalent bioconjugation of antibodies [22], thereby enhancing overall immunoprobe performance.

Herein, we propose modifying the surface of AgNPs with a PDA layer via DA self-polymerization to serve as a signal carrier. This strategy enhances the stability and dispersion of silver nanoparticles, preventing agglomeration while simultaneously improving their colorimetric capability. Building upon this, the conjugation of anti-AFB_1_ mAbs with Ag@PDA NPs as immunoprobes for LFIA detection of AFB_1_ enhances colorimetric signal intensity, shortens immunochromatographic analysis time, and improves detection sensitivity. This method enables rapid, sensitive, and accurate detection of AFB_1_ in maize, providing an innovative technical solution for maize quality control and AFB_1_ contamination prevention.

## 2. Results and Discussion

### 2.1. Principle of the LFIA

**Scheme 1:**
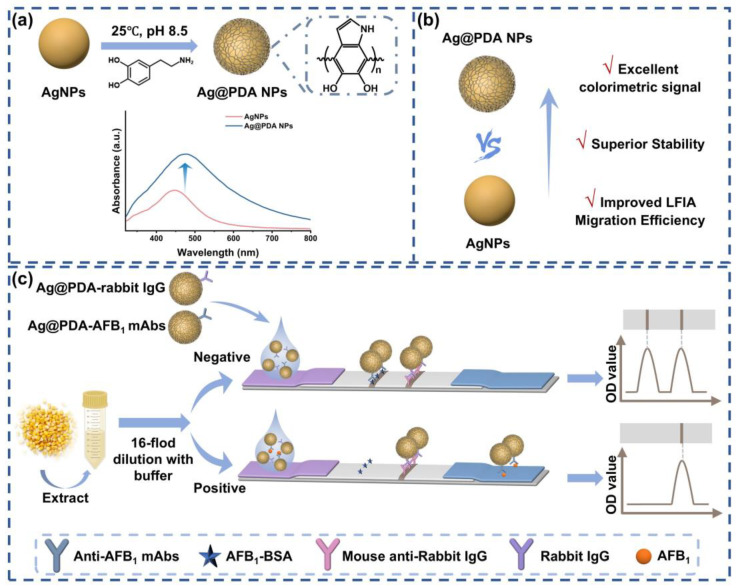
Schematic illustration of the LFIA. (**a**) Synthesis of Ag@PDA NPs and their corresponding spectra. (**b**) Illustration of the enhanced performance of Ag@PDA NPs compared to bare AgNPs. (**c**) Principle of AFB_1_ detection in negative and positive maize samples using Ag@PDA probes.

The principle of the LFIA is depicted in Scheme 1. Ag@PDA NPs were synthesized by first obtaining 79.2 nm AgNPs via the seed growth method, followed by the oxidation and self-polymerization of dopamine on the AgNP surface, where AgNPs serve as the core and PDA constitutes the outer shell (Scheme 1a). The Ag@PDA NPs obtained after coating AgNPs with PDA exhibit an excellent colorimetric signal, superior stability, and improved LFIA migration efficiency (Scheme 1b). The working principle of LFIA is illustrated in Scheme 1c. In the absence of AFB_1_ in the sample solution, the coated antigen on the T line captures the probes, producing a dark-yellow signal at the T line. Conversely, when AFB_1_ molecules are present in the sample solution, they compete with the coating antigen to bind to the antibodies on the probes. The signal intensity decreases as a result of this competition because fewer probes accumulate on the T line.

### 2.2. Characterization of Ag@PDA NPs

**Figure 1:**
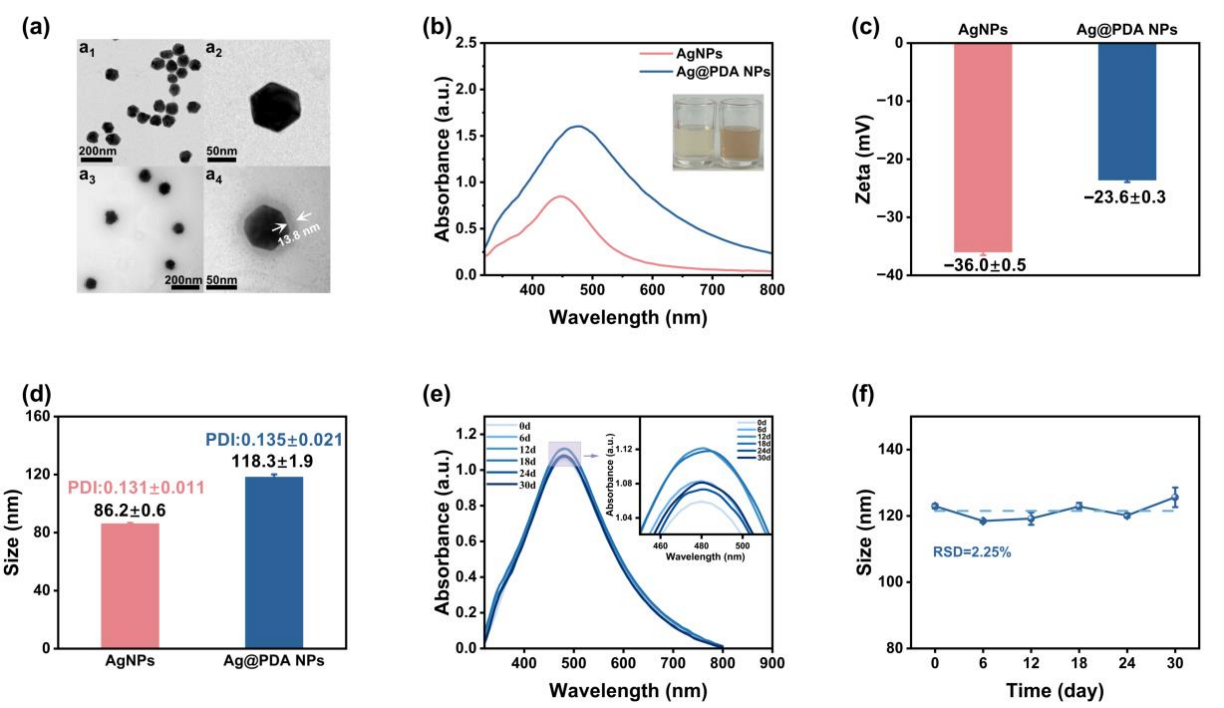
Characterization of AgNPs and Ag@PDA NPs. TEM images of (**a1**,**a2**) AgNPs, (**a3**,**a4**) Ag@PDA NPs. (**b**) UV-vis absorption spectra and digital photographs (inset) of AgNP and Ag@PDA NP solutions. (**c**) ζ-potential values and (**d**) hydrodynamic sizes of AgNPs and Ag@PDA NPs. (**e**) UV-vis absorption spectra and (**f**) hydrodynamic size of Ag@PDA NPs during 30 days of storage at room temperature. Vertical bars indicate standard deviation (*n* = 3).

Initially, AgNPs were fabricated via a seed-mediated growth approach. Subsequently, Ag@PDA NPs were produced through the in situ deposition of a PDA layer on AgNPs to form a core–shell structure. The morphology and size of the synthesized AgNPs and Ag@PDA NPs were characterized using TEM. The morphology and size of the synthesized AgNPs and Ag@PDA NPs were characterized by randomly analyzing 50 individual nanoparticles from TEM images. The TEM images in Figure 1a illustrate that the average diameters of AgNPs (Figure 1(a1)) and Ag@PDA NPs (Figure 1(a3)) were 79.2 and 106.8 nm, respectively. High-resolution TEM images of AgNPs in Figure 1(a2) and Ag@PDA NPs in Figure 1(a4) reveal that the average thickness of the PDA layer was 13.8 nm.

UV-vis spectroscopy was used to record the plasmonic properties of the nanoparticles. As displayed in Figure 1b, the AgNPs exhibited a sharp characteristic LSPR peak near 450 nm. Upon PDA coating, the LSPR peak of Ag@PDA NPs redshifted to approximately 475 nm, accompanied by a broadening of the peak width. Notably, the overall optical absorbance intensity at the peak wavelength increased by approximately 90% compared to bare AgNPs, attributed to the increased effective particle size and the additional absorption contribution of the PDA shell. The digital photos of the AgNP and Ag@PDA NP aqueous solutions in the inset of Figure 1b show that the color of the AgNP solution transformed from light yellow to dark-yellow after the PDA layer deposition. The as-prepared AgNPs and Ag@PDA NPs exhibited a uniform spherical shape. The evolution of ζ-potential values and hydrodynamic size (D_H_) in Figure 1c,d (based on *n* = 3 independent measurements) verified the successful synthesis of AgNPs and Ag@PDA NPs. All PDI values were below 0.2, indicating excellent monodispersity of the nanoparticles. The stability of Ag@PDA NPs was investigated by storing them in ultrapure water at room temperature for a month, with measurements conducted in triplicate (*n* = 3) at specific intervals. The results show that the absorption spectrum (RSD = 2.37%) and D_H_ value (RSD = 2.25%) of the Ag@PDA NPs are relatively stable. (Figure 1e,f). These findings indicate that the polydopamine layer improves the molar extinction ability and storage stability of Ag@PDA NPs. Additionally, the characterization of colloidal gold is shown in Figure S1 of the Supplementary Materials.

### 2.3. Construction and Optimization of LFIA Sensor

**Figure 2:**
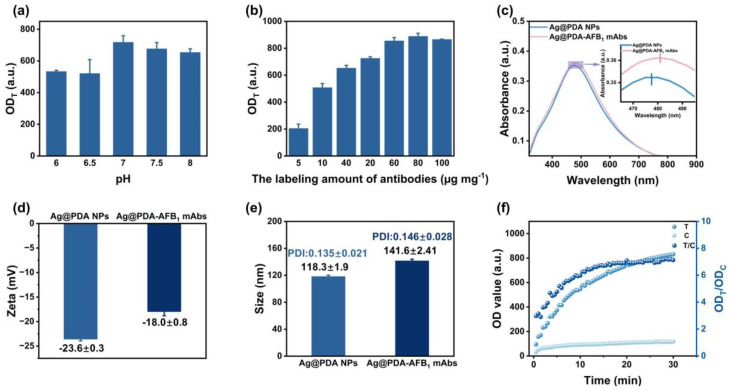
Optimization of LFIA parameters and characterization of Ag@PDA-AFB_1_ mAbs probes. Optimization of (**a**) pH and (**b**) antibody labeling amount. (**c**) UV-vis absorption spectra, (**d**) ζ-potential values, and (**e**) hydrodynamic sizes between Ag@PDA NPs and Ag@PDA-AFB_1_ mAbs. (**f**) Immunoreaction kinetic curves of T line, C line, and T/C (OD_T_/OD_C_) over time during the Ag@PDA-LFIA run. Vertical bars indicate standard deviation (*n* = 3).

Owing to its outstanding colorimetric performance, the anti-AFB_1_ mAbs-labeled Ag@PDA (Ag@PDA-AFB_1_ mAbs) was employed as the probe to construct LFIA. The Ag@PDA-AFB_1_ mAbs were prepared by coupling the anti-AFB_1_ mAbs on the surface of Ag@PDA NPs via electrostatic interactions. The labeling pH condition and amount of mAbs were optimized by recording the colorimetric signal intensity of Ag@PDA-AFB_1_ mAbs on the T line. As displayed in Figure 2a,b, the maximum colorimetric signal was achieved at a pH of 7.0 and a saturation labeling amount of 80 μg mg^−1^. Preparation methods for the colloidal gold or AgNP probes and optimization of their antigen dosage are detailed in Figure S2 and Tables S1 and S2 of the Supplementary Materials. Meanwhile, the increased redshifted absorption spectrum (Figure 2c), zeta (ζ) potential (Figure 2d), and hydrodynamic diameter (Figure 2e) of Ag@PDA-AFB_1_ mAbs verified the successful binding of mAbs to the Ag@PDA NPs surface, confirming effective bioconjugation.

The labeling amount of antibodies, coating antigen concentration, and probe loading were optimized via orthogonal array experiments to maximize LFIA detection performance. As summarized in Table 1, the optimal parameters were a T-line coating of 1 mg mL^−1^ AFB_1_-BSA conjugate, 20 μg mg^−1^ anti-AFB_1_ mAbs conjugated onto the surface of Ag@PDA, and 4.5 μL of Ag@PDA-AFB_1_ mAbs probes.

The optimal readout time of LFIA was evaluated by analyzing the immunological kinetic curve, which was determined by recording the intensities of the T line, C line, and T/C changes in a continuous 30 min running time in PBS (0.01 M, pH 7.4). Figure 2f shows that the T/C ratio stabilized after a 17 min runtime in PBS. Therefore, an immunoreaction time of 17 min for PBS is required to ensure the reproducibility of the LFIA for the quantitative analysis of AFB_1_. The AgNPs probes required a 25 min equilibration time to reach binding saturation on the nitrocellulose membrane, with detailed kinetic profiles provided in Figure S3 of the Supplementary Materials. The lateral flow velocity increased by 32% (*p* < 0.01) due to the enhanced dispersion and hydrophilicity of PDA-modified silver nanoparticles, effectively preventing agglomeration and hydrophobicity [23] from interfering with the chromatography process.

### 2.4. Evaluation of the Detection Performance of LFIA

**Figure 3:**
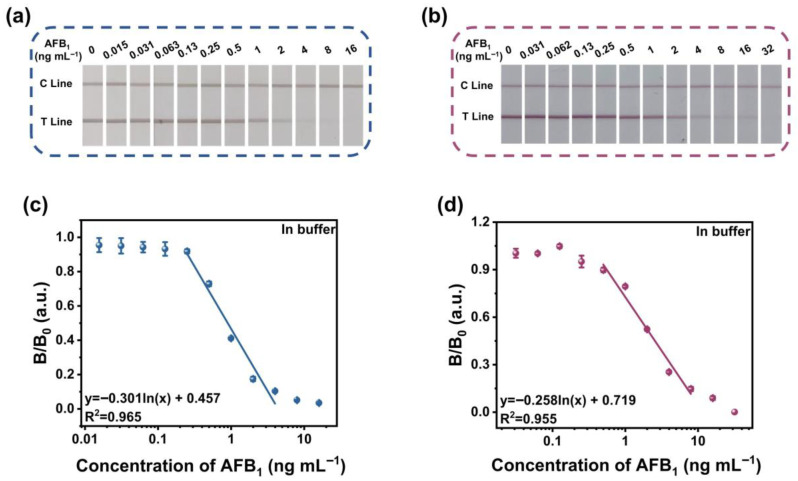
Comparison of detection performance between Ag@PDA-LFIA and AuNPs-LFIA. Digital photographs showing the visual detection of AFB_1_ using (**a**) Ag@PDA-LFIA and (**b**) AuNPs-LFIA strips. Standard calibration curves for quantitative AFB_1_ analysis using (**c**) Ag@PDA-LFIA and (**d**) AuNPs-LFIA. Vertical bars indicate standard deviation (*n* = 3).

The effectiveness of Ag@PDA-LFIA was assessed under ideal circumstances by identifying various AFB_1_ concentrations. As shown in Figure 3a,b, for Ag@PDA-LFIA and AuNPs-LFIA, the T-line color intensity was significantly weaker than that of the negative control at 0.5 ng mL^−1^ and 1 ng mL^−1^, respectively. Therefore, the corresponding visual limit of detection (vLOD) of Ag@PDA-LFIA was 0.5 ng mL^−1^, with a cut-off value of 16 ng mL^−1^. Compared to AuNPs-LFIA (vLOD: 1 ng mL^−1^; cut-off value: 32 ng mL^−1^), both results were 2 times lower. Concurrently, as the concentration of AFB_1_ increased, the dark grayish-yellow band at the T line of Ag@PDA-LFIA and the purple-red band at the T line of AuNPs-LFIA both gradually weakened. The standard calibration curve of LFIA for AFB_1_ quantitative detection was obtained by examining standard solutions of AFB_1_ at different concentrations. Figure 3c,d show the corresponding linear relationships between signal intensity and AFB_1_ concentration. For Ag@PDA-LFIA and AuNPs-LFIA, the linear regression equations are *y*_1_ = −0.301ln(*x*) + 0.457 and *y*_2_ = −0.258ln(*x*) + 0.719, respectively, where *y* is the B/B_0_, and *x* is the concentration of AFB_1_. The linear ranges were 0.25–4 ng mL^−1^ and 0.5–8 ng mL^−1^, respectively. Compared to AuNPs-LFIA (0.50 ng mL^−1^), Ag@PDA-LFIA had an LOD of 0.23 ng mL^−1^, which was 2.17 times lower.

**Figure 4:**
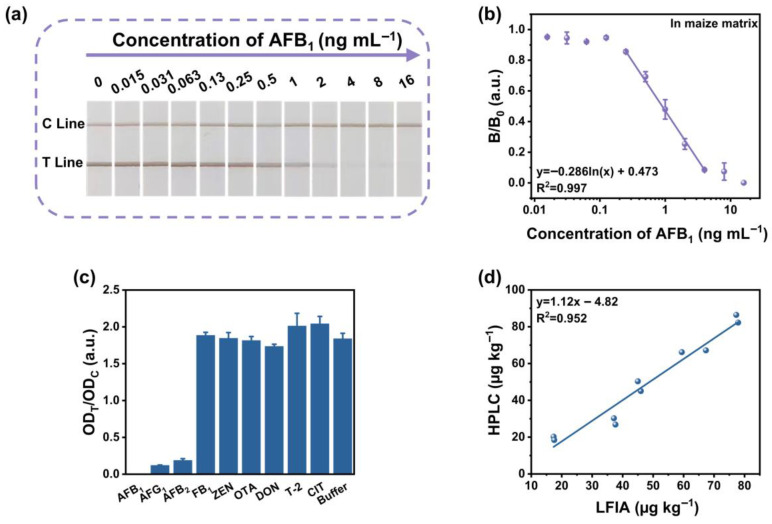
Validation of the Ag@PDA-LFIA assay in maize samples. (**a**) Digital photographs showing visual qualitative analysis of AFB_1_ in the maize sample. (**b**) Matrix-matched standard calibration curve for quantitative AFB_1_ analysis in maize extract. (**c**) Specificity evaluation of the LFIA sensor against common co-contaminating mycotoxins (AFB_1_, AFB_2_, and AFG_1_ at 20 ng mL^−1^; others at 200 ng mL^−1^). (**d**) Correlation analysis between the developed Ag@PDA-LFIA method and HPLC for AFB_1_ detection in artificially spiked maize samples. Vertical bars indicate standard deviation (*n* = 3).

Furthermore, the selectivity of the developed LFIA was assessed by analyzing common mycotoxins, including the analogs of aflatoxins (AFG_1_ and AFB_2_) and other mycotoxins such as FB_1_, ZEN, DON, OTA, T-2, and CIT. Figure 4c shows that AFG_1_ and AFB_2_ had some cross-reactivity with anti-AFB_1_ mAbs, while FB_1_, ZEN, DON, OTA, T-2, and CIT showed negligible cross-reactivity with the developed LFIA.

### 2.5. Matrix Effects and Determination of AFB_1_ in Maize Samples

To validate the quantitative capability of the developed LFIA for real maize samples, a matrix-matched calibration curve was established using AFB_1_-fortified maize matrices. By plotting standard curves for matrices at different dilution levels (Figure S4 in Supplementary Materials) and calculating the LOD and SSE values, Table S3 in Supplementary Materials shows that the LOD, R^2^, and SSE of the maize matrix diluted 16-fold are closest to those of the blank solution. This indicates that the matrix extract has minimal interference on the Ag@PDA-LFIA assay at this dilution factor. For actual testing, the maize samples were extracted with a methanol–water solution (70:30, *v*/*v*) at a ratio of 1:4 (*w*/*v*), and then diluted 16-fold for quantitative analysis. This dilution protocol mitigated matrix interference from maize-derived phenolic compounds, which impair antigen–antibody binding. Figure 4a shows a photograph of AFB_1_ detection in maize. The intensity of the color on the T line was visibly weaker than that of the negative control at the concentration of 0.5 ng mL^−1^. Therefore, the corresponding visual limit of detection (vLOD) was 0.5 ng mL^−1^, with a cut-off value of 16 ng mL^−1^. As shown in Figure 4b, the dilution series enabled the construction of AFB_1_ matrix calibration curves for the detection of real maize samples. The developed LFIA exhibited good linearity with the AFB_1_ concentration in the maize extract, varying from 0.25 ng mL^−1^ to 4 ng mL^−1^. The regression equation could be fitted as *y* = −0.286 ln(*x*) + 0.473 (R^2^ = 0.997), and the LOD was calculated as 0.22 ng mL^−1^ in maize extract (14.08 μg kg^−1^ in maize).

Furthermore, the recoveries and coefficient of variation (CV) for both intra-assay (three assays conducted within a day) and inter-assay (three assays at each day within three consecutive days) were analyzed in order to assess the accuracy and precision of the strip. The fortified AFB_1_ concentrations in maize samples were set at 20, 60, and 160 μg kg^−1^. Table 2 shows that the AFB_1_ recovery ranged from 95.70% to 119.28%, with the CVs less than 13.03%. These results demonstrated that the suggested LFIA for AFB_1_ quantitative detection in actual maize samples had adequate accuracy and precision.

Method trueness was further verified by comparing the LFIA results with high-performance liquid chromatography (HPLC). The linear regression equation between Ag@PDA-LFIA and HPLC was y = 1.12x − 4.82, with R^2^ = 0.952, demonstrating a strong correlation (Figure 4d), which confirmed the method’s reliability for practical applications.

## 3. Conclusions

This study establishes a robust on-site screening platform for AFB_1_ in maize—characterized by excellent probe stability, high recovery in complex matrices, and good analytical precision—by leveraging polydopamine-functionalized silver nanoparticles (Ag@PDA NPs) as advanced signal probes. These probes enhanced sensitivity compared to conventional probes through intensified plasmonic signals and fortified performance. The developed LFIA achieved an LOD of 0.22 ng mL^−1^ in maize extract (14.08 μg kg^−1^ in maize), which is significantly lower than stringent regulatory limits (20 μg kg^−1^ for maize and maize products in China; 20 μg kg^−1^ for maize feed in the EU) and represents a substantial improvement over traditional colloidal gold-based LFIAs. The spiked recovery rate of the developed LFIA ranges from 95.70% to 119.28%. The slightly high recoveries might be attributed to the matrix effect. Nevertheless, the low CV value (<13.03%) confirms that this method has excellent reliability and reproducibility. This platform demonstrates significant potential for rapid quantitative screening of mycotoxins and other food contaminants, with inherent adaptability to multiplexed detection systems.

## 4. Materials and Methods

### 4.1. Materials and Equipment

Silver nitrate (AgNO_3_), tannic acid (C_76_H_52_O_46_), trisodium citrate (C_6_H_5_Na_3_O_7_·2H_2_O), casein, bovine serum albumin (BSA), and hydrogen tetrachloroaurate trihydrate (HAuCl_4_·3H_2_O) were obtained from Sigma–Aldrich Chemical (St. Louis, MO, USA). Dopamine hydrochloride was provided by Aladdin Corp. (Shanghai, China). Aflatoxin B_1_ (AFB_1_), aflatoxin B_2_ (AFB_2_), aflatoxin G_1_ (AFG_1_), fumonisin B_1_ (FB_1_), zearalenone (ZEN), ochratoxin A (OTA), deoxynivalenol (DON), T-2 toxin (T-2), and citrinin (CIT) were acquired from Huaan Magnech Co., Ltd. (Beijing, China). Other analytical-grade chemicals were acquired from Aladdin Corp. (Shanghai, China) or Sinopharm Chemical. Sample pads, nitrocellulose (NC) membrane (CN 95), and absorbent pads were obtained from Shanghai Wenxin Jie Biotechnology Co., Ltd. (Shanghai, China). Polyvinyl chloride (PVC) pads were from Shanghai Jinbiao Biotechnology Co., Ltd. (Shanghai, China). AFB_1_-BSA and anti-AFB_1_ monoclonal antibody (anti-AFB_1_ mAbs) ascites were prepared by Wuxi Jieshengjiekang Co., Ltd. (Wuxi, China). Mouse anti-rabbit IgG and rabbit IgG were supplied by Beijing Solarbio Science & Technology Co., Ltd. (Beijing, China). All experiments were performed using distilled water purified by a Milli-Q system (ρ = 18.2 MΩ·cm) from Millipore Co. (Bedford, MA, USA).

DLS measurements were performed with a ZS90 particle size analyzer manufactured by Malvern Instruments Ltd. (Malvern, UK). High-resolution TEM images were captured using a JEOL JEM 2100 microscope (Tokyo, Japan). UV-vis absorption spectra were measured employing a Hitachi U-3900 spectrophotometer (Hitachi High-Technologies Corporation, Tokyo, Japan). The BioDot XYZ 3060 platform was supplied by BioDot Inc. (Irvine, CA, USA). Additionally, an automatic programmable cutter was procured from Shanghai Jinbiao Biotechnology Co., Ltd. (Shanghai, China), and the lateral flow strip reader was sourced from Feng Hang Technology Co., Ltd. (Hangzhou, China).

### 4.2. Synthesis of AgNPs and Ag@PDA NPs

AgNPs were synthesized using the seed growth method [24]. Briefly, 1 mL of 25 mM AgNO_3_ was added to 100 mL of a boiling aqueous solution (5 mM sodium citrate, 0.1 mM tannic acid) under stirring, resulting in an instantaneous transition to a bright yellow color. After seed preparation, 19.5 mL of the solution was discarded, and the residual seed dispersion was heated to 90 °C. A 19.5 mL growth solution, consisting of ultrapure water with 500 µL of 25 mM sodium citrate, 1.5 mL of 2.5 mM tannic acid, and 1 mL of 25 mM AgNO_3_, was then added. The mixture was incubated at 90 °C for 30 min to complete one growth cycle. This growth process was repeated for 14 successive cycles without intermediate cooling intervals. The number of cycles was selected based on prior empirical optimization to achieve a target core size of approximately 80 nm, which yielded optimal optical properties for subsequent PDA coating. After 14 successive cycles of this growth process, the final AgNP solution was prepared. To ensure method robustness, the reproducibility of synthesis was verified across three independent batches, showing consistent UV-vis spectral profiles and hydrodynamic diameters.

Ag@PDA NPs were prepared via coating the surface of AgNPs with a polydopamine layer. In brief, 5 mL of the aforementioned synthesized AgNP solution was centrifuged at 4450× *g* for 10 min. Then, the obtained pellet was redispersed in 9 mL of TRIS-HCl buffer (0.05 M, pH 8.5). Subsequently, 1 mL of 1 mg mL^−1^ dopamine solution was added to the mixture. After stirring for 8 h at room temperature, the color of the obtained Ag@PDA NPs colloid turned dark grayish-yellow. After centrifugation at 10,010× *g* for 10 min, the obtained Ag@PDA NPs were redispersed in 1 mL of ultrapure water and stored for use.

### 4.3. Preparation of Ag@PDA Probes

To establish a rabbit IgG-based independent control system, where the C-line signal production depends solely on the sample matrix and the inherent heterogeneity of the test strip—independent of probe quantity or target analyte concentration—we utilized a ratiometric T/C signal to ensure high reliability and stability [25]. Ag@PDA was conjugated with anti-AFB_1_ mAbs and rabbit IgG via electrostatic interactions to serve as detection and control probes, respectively [26]. Briefly, an optimized concentration of anti-AFB_1_ mAbs or 0.24 µg of rabbit IgG was added to 500 µL of 0.01 M PB (pH 7.0 for anti-AFB_1_ mAbs; pH 7.5 for rabbit IgG) containing 20 µg of Ag@PDA NPs. The mixture was allowed to react gently for 0.5 h. Subsequently, an equal volume of casein blocking solution (3%, *w*/*v*) was added and incubated for 1.5 h to passivate residual active sites. The resulting Ag@PDA-AFB_1_ mAbs or Ag@PDA-rabbit IgG conjugates were isolated via centrifugation (4450× *g*, 4 °C, 10 min) and resuspended in 50 µL of PB (0.01 M, pH 7.4) containing 25% sucrose, 0.1% casein, and 0.1% sodium azide, then stored at 4 °C. Successful conjugation and blocking were confirmed by a characteristic redshift in the UV-Vis absorbance spectrum, an increase in zeta potential, and an expanded hydrodynamic diameter relative to the unconjugated Ag@PDA NPs.

### 4.4. Construction of LFIA Sensor

The LFIA sensor was fabricated following a previously reported protocol with minor modifications [27]. The LFIA strip was composed of three functional components: nitrocellulose (NC) membrane, sample pad, and absorbent pad. It was crucial to pre-treat the sample pad to avoid nonspecific binding and guarantee the repeatability of the tests. The sample pads were immersed in PBS (0.05 M, pH 7.4) supplemented with 1.0% (*w*/*v*) BSA, 0.5% (*v*/*v*) Tween-20, and 0.05% (*w*/*v*) sodium azide for 30 s, followed by drying at 60 °C for 12 h before storage.

As a novel probe, the signal intensity of Ag@PDA probes at the test line—directly correlating with probe accumulation—required optimization for visual assessment. The amount of conjugated antibody, the AFB_1_-BSA coating density on the T line, and the probe dosage were identified as three critical factors affecting detection sensitivity. These were optimized through a three-factor, three-level orthogonal experiment (details presented in Section 2.3), ultimately selecting the parameter combination yielding both high T line intensity and high inhibition rate. Based on this optimization, the NC membrane was sprayed with AFB_1_-BSA and mouse anti-rabbit IgG solution (0.5 mg mL^−1^) at dispensing densities of 0.6 μL cm^−1^ for the T and C lines (the two lines were separated by 5 mm), respectively. For quality control during fabrication, only strips exhibiting visually uniform T and C lines across the batch were selected for use. Inter-strip reproducibility was assessed, and batches yielding a coefficient of variation (CV) of >10% in signal intensity for blank samples were rejected. The NC membrane was dried at 37 °C for 12 h. The construction was sliced into 3.9 mm broad strips after the NC membrane, sample pad, and absorbent pad were laminated onto the plastic backing plate. For later usage, each strip was placed into a stiff plastic cassette with a reading window and sample well, then sealed in a plastic bag with desiccant gel.

### 4.5. Quantitative AFB_1_ Detection Using LFIA Sensor

AFB_1_ quantitative analysis was performed by detecting a series of diluted AFB_1_ standard solutions using the LFIA sensor. First, 70 μL of standard AFB_1_ solution (0, 0.015, 0.031, 0.063, 0.13, 0.25, 0.5, 1, 2, 4, 8, and 16 ng mL^−1^) was incubated with 4.5 μL of Ag@PDA-AFB_1_ mAbs and 1 μL of Ag@PDA-rabbit IgG for 3 min, followed by adding the incubated mixture to the sample well of the LFIA strip. After a 17 min immunoreaction period, the colorimetric intensity of the T and C lines was measured using a customized strip reader. The standard curve was constructed by plotting the B/B_0_ ratios against the logarithm of AFB_1_ concentrations, where B and B_0_ stand for the T/C ratio of the sample and the T/C ratio of the negative control, respectively. The visual limit of detection (vLOD) refers to the minimum concentration at which the color intensity of the T line is visibly and distinctly weaker than that of the negative control [28,29]. The limit of detection (LOD) was determined as the AFB_1_ concentration corresponding to a 10% inhibition rate in the competitive binding reaction [30,31].

### 4.6. Specificity Evaluation of LFIA

To evaluate the specificity of the developed LFIA sensor, several structurally related mycotoxins (AFB_2_, AFG_1_) and unrelated mycotoxins (OTA, ZEN, DON, FB_1_) were tested. AFB_1_ was tested at 20 ng mL^−1^, structurally similar mycotoxins (AFB_2_ and AFG_1_) were tested at the same concentration (20 ng mL^−1^) to assess potential cross-reactivity, while structurally unrelated mycotoxins were tested at 10-fold higher concentrations (200 ng mL^−1^) to rigorously challenge assay specificity. The specificity of the method was evaluated by comparing the OD_T_/OD_C_ values obtained from different mycotoxins to those from blank buffer controls [32]. Each experimental condition was replicated three times independently.

### 4.7. Quantitative Detection of AFB_1_ in Maize Samples

Maize sample extract preparation and HPLC analysis complied with the specifications of GB 5009.22-2016 (Chinese National Food Safety Standard) [33]. Specifically, 5 g of maize flour was subjected to extraction using a 70:30 (*v*/*v*) methanol–water mixture with shaking for 20 min. Afterward, the mixture was centrifuged at 2500× *g* for 10 min, and the resulting supernatant was collected for subsequent use. Extracts were diluted 4- to 32-fold. A matrix-spiked calibration curve was plotted at each dilution concentration and compared with a buffer-based calibration curve to assess matrix effects. Matrix effect comparison across dilutions referenced the SSE (Signal Suppression/Enhancement) formula for matrix effect evaluation in chromatographic calibration curves [34].

SSE = (Slope of standard curve for matrix-matched solution/Slope of standard curve for standard solution) × 100%.

To assess the accuracy and precision of LFIA, the average recoveries and coefficient of variation (CV) of the strips were determined with AFB_1_-spiked samples, which were prepared by adding AFB_1_ at concentrations of 20, 60, and 160 μg kg^−1^ to AFB_1_-negative maize samples. All experiments were conducted three times.

To further validate the reliability and practicality of the Ag@PDA-LFIA detection method, five (*n* = 5) artificially spiked maize samples were tested, and the results were compared with established HPLC results through linear regression analysis.

### 4.8. Statistical Analysis

Data analysis and graphing were performed using Origin 2024. All experiments were carried out in triplicate, and data are expressed as mean ± standard deviation (SD) unless stated otherwise.

## Figures and Tables

**Table 1:** Results of the orthogonal experiment for optimizing key Ag@PDA-LFIA parameters: AFB_1_-BSA coating concentration, antibody labeling amount, and probe loading volume.

No.	The Concentration of AFB_1_-BSA (mg mL^−1^)	The Labeling Amounts of Antibodies (μg mg^−1^)	The Volume of Probes (μL)	The OD of T Line (Negative)	The Inhibition Rate (%) ^a^
1	0.5	10	1.5	129.1 ± 7.1	100.0
2	0.5	20	3	362.5 ± 34.8	88.4
3	0.5	40	4.5	702.7 ± 17.9	56.4
4	1	10	3	304 ± 9.3	89.4
5 *	1	20	4.5	665.5 ± 12.8	85.0
6	1	40	1.5	513.7 ± 28.0	82.6
7	1.5	10	4.5	507.3 ± 11.5	83.8
8	1.5	20	1.5	365.3 ± 23.9	100.0
9	1.5	40	3	766.9 ± 18.1	72.1

^a^ The inhibition rates are obtained from the 2 ng mL^−1^ AFB_1_-spiked sample. * The asterisk indicates the optimal detection conditions.

**Table 2:** Precision and accuracy of the Ag@PDA-LFIA sensor for spiked maize samples.

Spiked (μg kg^−1^)	Intra-Assay Precision ^a^			Inter-Assay Precision ^b^		
	Mean (μg kg^−1^)	Recovery (%)	CV (%)	Mean (μg kg^−1^)	Recovery (%)	CV (%)
20	19.59	97.96	0.88	19.14	95.70	10.76
60	65.02	108.37	5.85	71.57	119.28	13.03
160	161.23	100.77	1.84	159.09	99.43	9.82

^a^ Three assays were conducted within a day. ^b^ Three assays at each day within three consecutive days.

## Data Availability

The original contributions presented in this study are included in the article and Supplementary Material. Further inquiries can be directed to the corresponding author.

## Supplementary Materials

The following supporting information can be downloaded at https://www.mdpi.com/article/10.3390/toxins18030129/s1, Section S1: Synthesis of AuNPs; Section S2: Optimization of key parameters of AuNPs-LFIA and AgNPs-LFIA; Section S3: Process of HPLC; Figure S1. Characterization of AuNPs; Figure S2. Optimization of the labeling pH condition for the preparation of AuNPs-AFB_1_ mAbs probes, and AgNPs-AFB_1_ mAbs probes; Figure S3. Immunoreaction kinetic curves illustrating the changes in T-line intensity, C-line intensity, and the T/C (OD_T_/OD_C_) ratio over time during the AgNPs-LFIA run; Figure S4. Evaluation of maize matrix interference on AFB_1_ detection using Ag@PDA-LFIA at different dilution ratios; Table S1. Results of the orthogonal experiment for optimizing key AuNPs-LFIA parameters; Table S2. Results of the orthogonal experiment for optimizing key AgNPs-LFIA parameters; Table S3. Linear regression equation, LOD, Linearity (R^2^), and Signal Suppression/Enhancement (SSE) indices of the maize matrix evaluated at different dilution ratios.
