# Supplementary material for: Boosting Sensitivity, Stability, and Speed: A Polydopamine-Engineered Silver Nanoparticle Lateral Flow Immunoassay for Aflatoxin B1 in Maize
Source: Toxins (Basel). 2026 Mar 3;18(3):129. doi: 10.3390/toxins18030129 (PMC13030584; doi:10.3390/toxins18030129)
Supplement: Supplementary file 1 [file toxins-18-00129-s001.zip › toxins-4142624-supplementary.pdf]

Supplementary Materials: Boosting Sensitivity, Stability, and Speed: A Polydopamine-Engineered Silver Nanoparticle Lateral Flow Immunoassay for Aflatoxin B_1 in Maize

Xinge Mo, Shuhong Zhang, Zixuan He, Xiaoyang Li, Xiangmin Li, Yonghua Xiong and Hu Jiang

Content

**1.** Synthesis of AuNPs.	2
**2.** Optimization of key parameters of AuNPs-LFIA and AgNPs-LFIA.	2
**3.** Process of HPLC.	2
**Figure S1.** Characterization of AuNPs.	3
**Figure S2.** Optimization of the labeling pH condition for the preparation of AuNPs-AFB$_1$ mAbs probes and AgNPs-AFB$_1$ mAbs probes.	3
**Figure S3.** Immunoreaction kinetic curves illustrating the changes in T-line intensity, C-line intensity, and the T/C (OD_T/OD_C) ratio over time during the AgNPs-LFIA run.	4
**Figure S4.** Evaluation of maize matrix interference on AFB$_1$ detection using Ag@PDA-LFIA at different dilution ratios.	4
**Table S1.** Results of the orthogonal experiment for optimizing key AuNPs-LFIA parameters.	5
**Table S2.** Results of the orthogonal experiment for optimizing key AgNPs-LFIA parameters.	5
**Table S3.** Linear regression equation, LOD, Linearity (R^2), and Signal Suppression/Enhancement (SSE) indices of the maize matrix evaluated at different dilution ratios.	5

Experiment Section

1. Synthesis of AuNPs

AuNPs were synthesized using the trisodium citrate reduction method. Briefly, 99.5 mL of ultrapure water and 500 μL of 2% $HAuCl_4 \cdot 3H_2O$ solution were stirred and heated to boiling. Under continuous accelerated stirring, rapidly add 1.7 mL of freshly prepared trisodium citrate solution (1%, w/v). Maintain stirring at a constant speed for 20 min until the solution color stabilizes. Finally, the AuNPs solution was cooled to room temperature and stored at 4 °C.

2. Optimization of key parameters of AuNPs-LFIA and AgNPs-LFIA

For AuNPs-LFIA, to optimize the conjugation reaction, different volumes of K_2CO_3 (0.2 M) were added to the AuNPs solution to adjust the pH to 6, 6.5, 7, 7.5, and 8. Orthogonal experiments were conducted to optimize the AFB_1-BSA concentration on the T line, the anti-AFB_1 mAbs concentration, and probe usage, with concentrations of 0.5, 1, and 1.5 mg mL^{-1}; 2, 4, and 8 μg mL^{-1}; and 1.5, 3, and 4.5 μL, respectively. All experiments were repeated three times.

For AgNPs-LFIA, to optimize the conjugation reaction, 300 μL of AgNPs solution was centrifuged at 4450 × g for 10 min, then redissolved in 300 μL of PB (0.01M) at pH 6, 6.5, 7, 7.5, and 8, respectively. Orthogonal experiments optimized the AFB_1-BSA concentration on the T line, the anti-AFB_1 mAbs concentration, and probe usage, with concentrations of 0.5, 1, and 1.5 mg mL^{-1}; 5, 10, and 20 μg mg^{-1}; and 0.5, 1, and 2 μL, respectively. All experiments were replicated three times.

3. Process of HPLC

AFB_1 samples were analyzed using HPLC-UV. Separation was performed with an Agilent Zorbax Eclipse Plus C18 column (4.6 mm × 250 mm, 5 μm). AFB_1 detection parameters included a mobile phase of water-acetonitrile (45:55), an injection volume of 20 mL, a flow rate of 0.6 mL min^{-1}, a column temperature of 40 °C, and UV detection at 365 nm.

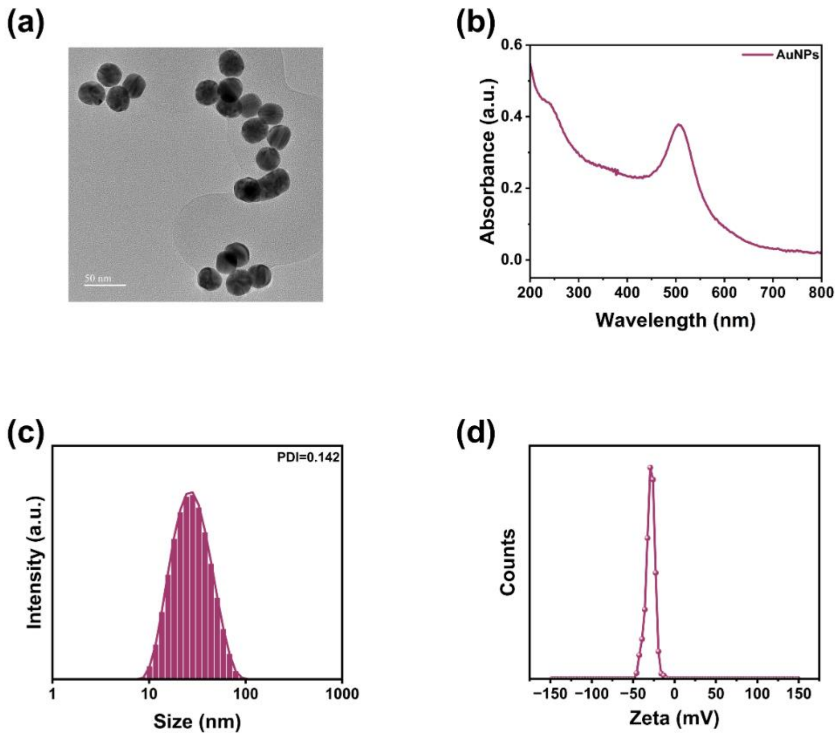

Figure S1. Characterization of AuNPs. (a) TEM image, (b) UV-vis absorption spectrum, (c) hydrodynamic size, (d) ζ-potential value.

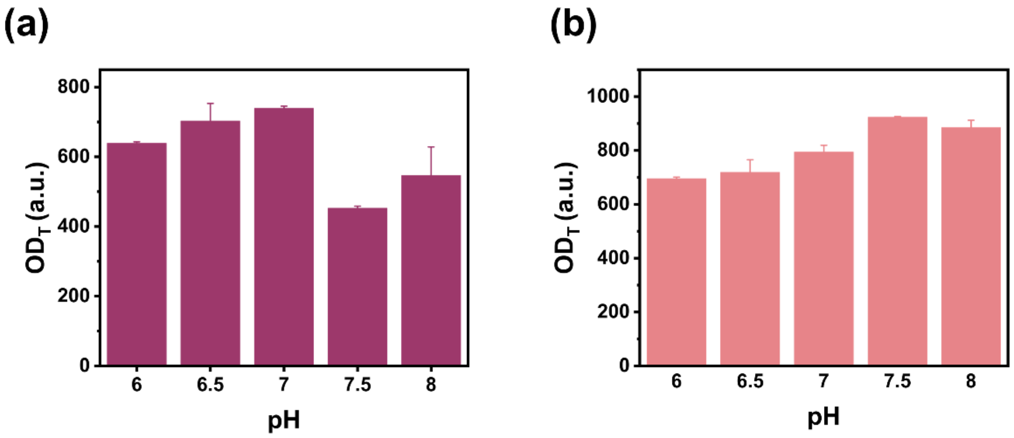

Figure S2. Optimization of the labeling pH condition for the preparation of (a) AuNPs-AFB_1 mAbs probes, and (b) AgNPs-AFB_1 mAbs probes.

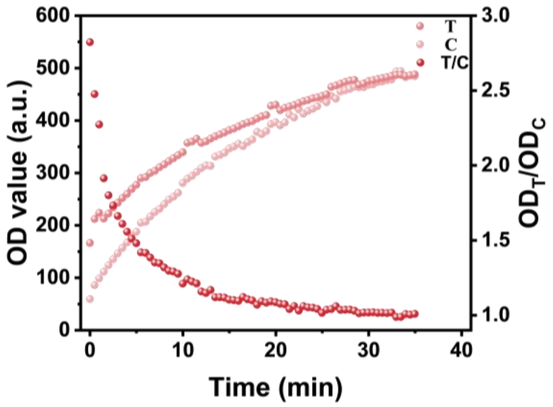

Figure S3. Immunoreaction kinetic curves illustrating the changes in T-line intensity, C-line intensity, and the T/C (OD_T/OD_C) ratio over time during the AgNPs-LFIA run.

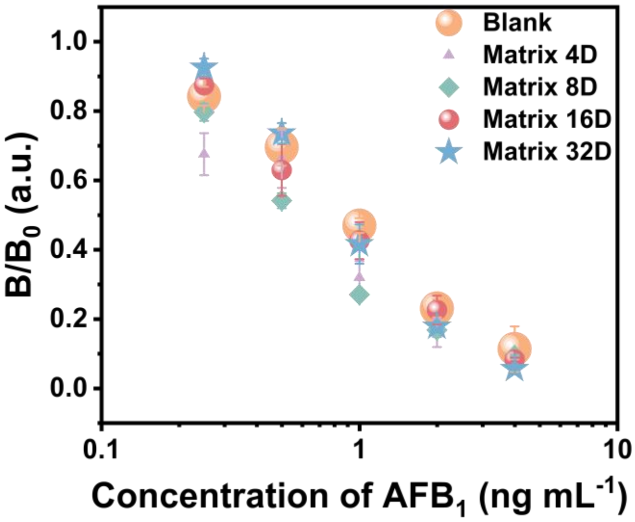

Figure S4. Evaluation of maize matrix interference on AFB_1 detection using Ag@PDA-LFIA at different dilution ratios. Vertical bars indicate standard deviation ($n = 3$).

Table S1. Results of the orthogonal experiment for optimizing key AuNPs-LFIA parameters: AFB_1-BSA coating concentration, antibody labeling amount, and probe loading volume.

No.	The concentration of AFB_1-BSA (mg mL^{-1})	The labeling amounts of antibodies (μg mL^{-1})	The volume of probes (μL)	The OD of T line (negative)	The inhibition rate (%) [a]
1	0.5	2	1.5	132.5 ± 4.8	100.0
2	0.5	4	3	450.4 ± 13.6	70.4
3	0.5	8	4.5	597.4 ± 14.2	61.1
4	1	2	3	348.6 ± 3.5	87.9
5	1	4	4.5	778.7 ± 19.0	59.6
6	1	8	1.5	451.2 ± 12.3	88.0
7	1.5	2	4.5	363.6 ± 11.0	87.0
8	1.5	4	1.5	317.5 ± 9.3	68.7
9*	1.5	8	3	613.1± 8.5	68.2

[a] The inhibition rates are obtained from the 2 ng mL^{-1} AFB_1-spiked sample.

* The asterisk indicates the optimal detection conditions.

Table S2. Results of the orthogonal experiment for optimizing key AgNPs-LFIA parameters: AFB_1-BSA coating concentration, antibody labeling amount, and probe loading volume.

No.	The concentration of AFB_1-BSA (mg mL^{-1})	The labeling amounts of antibodies (μg mg^{-1})	The volume of probes (μL)	The OD of T line (negative)	The inhibition rate (%) [a]
1	0.5	5	0.5	112.5 ± 4.6	100.0
2	0.5	10	1	339.5 ± 43.1	86.8
3	0.5	20	2	813.5 ± 25.3	40.4
4	1	5	1	238.0 ± 28.8	100.0
5*	1	10	2	558.8 ± 28.1	82.2
6	1	20	0.5	521.6 ± 45.2	67.3
7	1.5	5	2	370.1 ± 18.4	86.0
8	1.5	10	0.5	365.3 ± 49.8	84.3
9	1.5	20	1	684.8 ± 60.3	52.6

[a] The inhibition rates are obtained from the 1 ng mL^{-1} AFB_1-spiked sample.

* The asterisk indicates the optimal detection conditions.

Table S3. Linear regression equation, LOD, Linearity (R^2), and Signal Suppression/Enhancement (SSE) indices of the maize matrix evaluated at different dilution ratios.

Dilution ratio	Linear regression equation	LOD (ng mL^{-1})	Linearity (R^2)	SSE
blank	$y=-0.297\ln(x)+0.457$	0.23	0.989	—
4	$y=-0.271\ln(x)+0.401$	0.16	0.904	91.25
8	$y=-0.311\ln(x)+0.336$	0.16	0.969	104.71
16	$y=-0.310\ln(x)+0.431$	0.22	0.998	104.38
32	$y=-0.370\ln(x)+0.436$	0.29	0.993	124.58
